# PuraBond® in Robot-Assisted Radical Prostatectomy (RARP): Exploring a Novel Haemostatic Strategy Using Self-Assembling Peptides

**DOI:** 10.7759/cureus.95404

**Published:** 2025-10-25

**Authors:** Karthika Kalissery Biju Chandrasekhar, Sakshi Rajain, Samuel John Davies, Jonathan Noël

**Affiliations:** 1 Urology, Guy's and St Thomas' National Health Service (NHS) Foundation Trust, London, GBR

**Keywords:** haemostasis, prostate cancer (pca), purabond®, robot-assisted radical prostatectomy, self-assembling peptide

## Abstract

Introduction

Haemostasis is critical during and after robot-assisted radical prostatectomy (RARP) for prostate carcinoma. While RARP offers superior outcomes compared to open and laparoscopic surgery, achieving reproducible intra- and postoperative bleeding control is essential for all approaches. Conventional methods of haemostasis in the pelvis and the prostate bed include diathermy and sutures, which may be insufficient in some cases. This report evaluates the use of PuraBond® (3D Matrix Ltd., Tokyo, Japan), a self-assembling peptide (SAP) haemostatic agent, during RARP as a novel method.

Materials and methods

We conducted a retrospective case series of 10 patients who underwent RARP at a tertiary National Health Service (NHS) teaching hospital between January and March 2025. Data on patient demographics, surgical approach, and follow-up outcomes were collected from institutional records. Variables recorded included patient age, perioperative parameters such as nerve-sparing status, estimated blood loss, and transfusion requirements. Overall complications and readmissions within 90 days were recorded. Urinary function was assessed using pre-operative and post-operative International Prostate Symptom Score (IPSS) scores, with continence defined as the absence of pad use. Erectile function (EF) was evaluated using preoperative and postoperative International Index of Erectile Function (IIEF) scores. The primary endpoints were intraoperative complications, especially postoperative haemostasis. Secondary endpoints included catheter removal success and urinary outcomes on follow-up.

Results

Ten patients (age range: 51-76 years, median age: 66 years) underwent RARP with intraoperative application of PuraBond®. No intraoperative complications were observed, and PuraBond® achieved effective haemostasis in all cases. No patients experienced postoperative bleeding or required a blood transfusion. Trial without catheter (TWOC) was successful in all patients, typically at the first follow-up visit (median: 13 days; range: 12-15 days). At the initial postoperative review (mean: 12 days), most patients reported either mild or no urinary incontinence. Outcomes at the second follow-up (mean: 48 days) demonstrated continued recovery with low complication rates maintained. No readmissions nor adverse events were reported within the 90-day postoperative period.

Conclusion

This study presents the first reported case series evaluating PuraBond® as an adjunct haemostatic agent in RARP. The results suggest consistent haemostatic efficacy and favourable early postoperative outcomes across a patient cohort. Prospective controlled studies are warranted to compare PuraBond® with alternative methods of haemostasis.

## Introduction

Radical prostatectomy (RP) has been the standard surgical option for clinically localised prostate cancer (PCa) [1], with a significant shift from open radical prostatectomy (ORP) to robot-assisted radical prostatectomy (RARP). Since 2001 [2], RARP has rapidly evolved and is becoming the primary surgical approach for prostatectomy in many countries [3].

The management of perioperative bleeding in prostatic surgery is challenging, with potential consequences including increased morbidity and lower patient satisfaction. A key advantage of robotic surgery is its ability to minimise intraoperative bleeding. A randomised trial found significantly lower intraoperative blood loss with RARP compared with ORP (median 250.0 vs 719.5 mL; P<0.001) [4]. This advantage contributes to faster recovery and reduced complication rates. RP via an open retropubic or robot-assisted approach will have a risk of significant blood loss; therefore, intraoperative haemostatic control is essential [5]. However, RARP also has its own limitations, owing to the impact on post-operative functional outcomes, particularly regarding erectile function and urinary continence.

Conventional haemostasis methods during RARP include thermal techniques (monopolar or bipolar diathermy), mechanical methods (clips and haemostatic sutures), and adjunctive haemostatic products, such as fibrin sealants or absorbable haemostats. These agents have prompted further exploration of haemostatic adjuncts to improve intraoperative bleeding control. Various agents, including fibrin glues, thrombin-based products, and absorbable haemostats, have been investigated [6,7].

PuraBond® (also known as PuraStat®; 3D Matrix Ltd., Tokyo, Japan) is a haemostatic agent composed of self-assembling peptides (SAPs), specifically the RADA16 sequence. Upon contact with blood, PuraBond® forms a gel-like matrix that acts as a mechanical barrier, encouraging clot stabilisation without interfering with the body's natural coagulation processes [8]. The chemically synthesised amino acids are bound together in a specific sequence at a 2.5% concentration in aqueous solution to make PuraBond® [9]. Unlike conventional haemostatic agents, PuraBond® forms a transparent peptide matrix that allows natural coagulation while maintaining visual clarity and may also support tissue healing, making it particularly suitable for delicate procedures such as RARP.

There are no specific contraindications to using self-assembling peptides (SAPs) like PuraBond® reported in peer-reviewed literature to date across surgical and endoscopic procedures. There is increasing evidence of its use in gastrointestinal, vascular, and urological surgery, supporting its use as a haemostatic adjunct without additional risk to a wide pathology of patients [10]. Specifically, PuraBond® has also demonstrated effectiveness in fields like colorectal surgery, where it has been shown to achieve high rates of initial haemostasis and low rates of rebleeding [11], and in cardiovascular surgery applications such as vascular anastomoses and suture-lines [12]. However, its use in urological procedures such as RARP is yet to be investigated. We aim to assess the safety and feasibility of PuraBond® for haemostasis and its impact on early recovery from RARP in a single-centre setting by a single surgeon.

## Materials and methods

This retrospective observational case series evaluates the use of PuraBond® during RARP. All procedures were carried out by a surgeon performing approximately 100 robotic cases annually, providing supervision of senior fellow surgeons. RARP was performed using Intuitive da Vinci Surgical System (Intuitive Surgical, Sunnyvale, CA, USA) X model between January-March 2025.

Surgical technique

**Figure 1:**
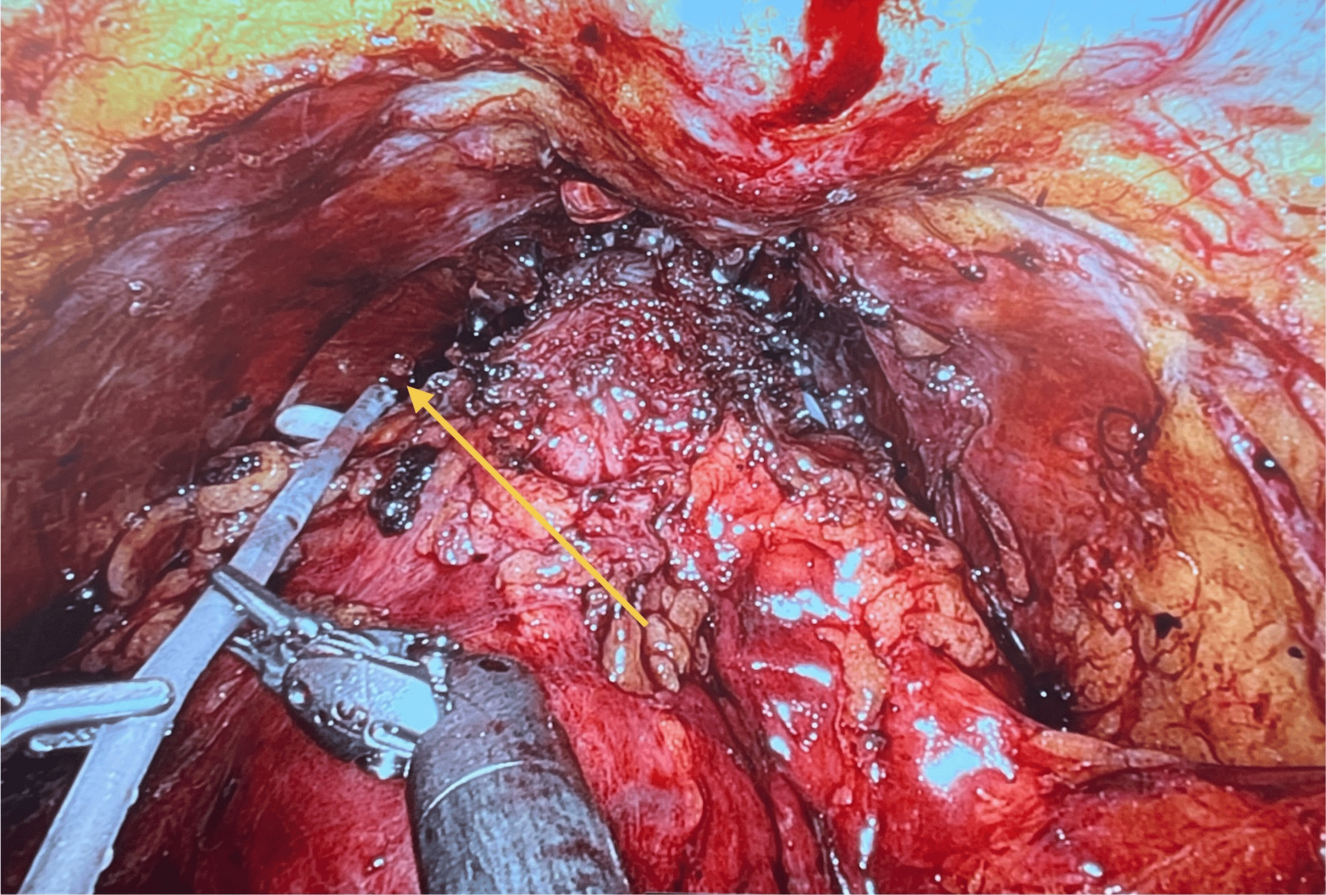
Intraoperative image Endoscopic view during robot-assisted radical prostatectomy following completion of the urethrovesical anastomosis, demonstrating the application of PuraBond® to the prostatic apex for haemostatic control.

After standard transperitoneal RARP with the multi-port da Vinci System, Initial haemostasis was achieved with Hem-o-lok® clips (HOLC) (Weck Surgical Instruments, Teleflex Medical, Durham, NC) and spiral polydioxanone (PDS; STRATAFIX®; Ethicon, Somerville, NJ) sutures. Five millilitres of PuraBond® were then instilled into the apex in all patients under direct vision through an applicator via the assistant’s port (Figure 1). Leak testing of the vesicourethral anastomosis was performed after urinary catheter insertion, followed by pelvic drain insertion.

Data collection

Patient demographics, surgical details, and follow-up outcomes were collected from institutional records and summarised without patient identifiers. Follow-up assessments included the first three postoperative follow-up consultations. Data on the urinary catheter removal status, post-operative PSA levels, specimen surgical margin status, complications, and hospital readmissions within 90 days were retrieved. Urinary function was evaluated by continence, defined by the number of pads used per day. Erectile function (EF) was evaluated using the International Index of Erectile Function (IIEF) preoperatively (baseline) and at the 3-month follow-up, within 90 days postoperatively.

Endpoints

Primary

The primary endpoint of this case series is the feasibility and safety of PuraBond® as a haemostatic agent during robot-assisted radical prostatectomy (RARP). This included intraoperative haemostasis, absence of postoperative bleeding, perioperative haemoglobin (Hb) change, postoperative drain output (millilitres), and the need for blood transfusion.

Secondary

Secondary endpoints included early functional recovery and postoperative outcomes. These comprised the success of trial without catheter (TWOC), urinary continence (no pad usage), erectile function (change in IIEF score), and early oncological control assessed by postoperative PSA levels and margin status. Additional measures were the incidence of intraoperative complications and the rate of readmissions or adverse events within 90 days (Table 1).

**Table 1:** Secondary endpoints: early functional recovery, oncological control, and postoperative outcomes

Surgery Date (Month/Year)	Nerve Spare	Haemoglobin (grams per litre) - Pre and Post-operative	Drain Output (millilitres)	Trial Without a Catheter (TWOC) Outcome	Three Month Follow-up Prostate Specific Antigen	International Index of Erectile Function - Pre (baseline) and Post-Operative (3months)	Number of Pads	Resection Margins
									01/2025	Left-None, Right>50%	156 /124	15	Yes	Undetectable	15/7	1 (Wet)	Clear
									01/2025	Left-None, Right-None	132/119	0	Yes	Undetectable	15/0	1 (Wet/Dry)	Circumferential Margin involved
02/2025	Left- Full, Right- Full	160/136	30	Yes	Undetectable	15/0	0	Clear
02/2025	Left>50%, Right>50%	114/91	10	Yes	0.85	15/17	1 (Wet/Dry)	Clear
02/2025	Left- Full, Right- Full	137/137	0	Yes	Undetectable	15/10	2 (Wet)	Clear
02/2025	Left-None, Right-None	116/91	0	Yes	Undetectable	15/5	7	Clear
02/2025	Left<50%, Right-None	135/116	20	Yes	Undetectable	15/5	0	Clear
03/2025	Left- Full, Right>50%	145/137	20	Yes	Undetectable	15/7	1 (Wet/Dry)	Clear
03/2025	Left- Full, Right- Full	132/112	0	Yes	Undetectable	15/8	0	Clear
03/2025	Left- Full, Right>50%	133/117	0	Yes	Undetectable	15/11	1 0(Wet/Dry)	Clear

## Results

No cases of primary or secondary haemorrhage were reported following the application of PuraBond® during RARP, indicating effective intraoperative haemostasis. None of the patients required a blood transfusion, further supporting the haemostatic efficacy of the agent. Additionally, there were no intraoperative complications, hospital re-attendances, or readmissions within the postoperative period, suggesting a favourable safety profile. These findings demonstrate that the use of PuraBond® was associated with a smooth perioperative course and minimal postoperative morbidity. Overall, the results suggest that PuraBond® can be safely utilised as an adjunct haemostatic agent in RARP without increasing the risk of complications or adverse events.

## Discussion

This series demonstrates that PuraBond® is a feasible haemostatic adjunct in RARP with consistent effectiveness across a cohort. With regards to peri-operative outcomes, intra-operative haemostasis was achieved in 100% of cases. There were no intraoperative complications, postoperative readmissions, or bleeding events observed within 90 days. Evidence from other surgical fields supports the haemostatic potential of PuraBond®. In urological surgery, particularly following HoLEP, PuraBond® has been shown to provide stable haemostasis, with no postoperative bleeding or readmissions up to 28 days post-procedure [8]. In head and neck surgery (transoral robotic surgery (TORS(JN1)), it eliminated both early and late postoperative haemorrhage in a 12-procedure series [13]. Further reports across tonsillectomy, GI endoscopic dissection, and comparative animal models demonstrate significantly lower rebleeding rates and reduced reliance on thermal coagulation when PuraBond® or related RADA16 agents were used [14].

An additional benefit is that PuraBond® is a transparent agent. Once applied to the operative field, it gives surgeons a clear visualisation of the prostatectomy bed to locate active bleeding points. An adjunct that is transparent and athermal, PuraBond® can protect nerve-spared tissue from trauma of diathermy or suturing [9].

Functional outcomes in our cohort were consistent with published data post-RARP: 80% of patients had only mild to moderate incontinence (≤1 pad/day), and 30% were pad-free at first follow-up. Erectile dysfunction was reported in the majority of patients, consistent with the early phase of follow-up post-RARP. The trajectory will continue to improve and be optimised through a structured survivorship pathway incorporating specialist andrology and functional rehabilitation [15].

Oncological outcomes showed PSA persistence on the first postoperative check in one case, with negative resection margins. These results suggest that PuraBond® may be a safe addition to standard haemostatic techniques in RARP, without compromising cancer control. The short-term follow-up, we have demonstrated, shows that PuraBond® is feasible to apply on the prostatic bed post-RARP. Further research involving larger prospective cohorts of patients and longer follow-up periods is necessary. Additionally, comparative analyses with alternative haemostatic agents would be of interest to the urology community. Therefore, the limitations of this series include the retrospective single-centre design, small sample size, and absence of a control group, all of which may limit the generalisability of the findings and make it difficult to draw definitive cause-and-effect conclusions. Additionally, the short duration of follow-up restricts assessment of long-term functional and oncological outcomes.

## Conclusions

Intraoperative application of PuraBond® during RARP appeared safe and feasible as a haemostatic adjunct, demonstrating satisfactory haemostasis without complications. However, given the retrospective nature of this study, absence of a control group, and limited sample size, these findings should be interpreted as preliminary and hypothesis-generating. Further prospective, randomised studies comparing PuraBond® with other established haemostatic techniques are warranted to better define its efficacy and clinical role in RARP.
